# Risk factors of respiratory tract infection in patients after surgery under general anesthesia and nursing countermeasures

**DOI:** 10.4314/ahs.v25i3.7

**Published:** 2025-09

**Authors:** Xudong Xu, Yuan Zhao

**Affiliations:** 1 Department of Anesthesia, Tianjin Hospital, Tianjin 300211, China; 2 School of Nursing, Tianjin University of Traditional Chinese Medicine, Tianjin 301617, China

**Keywords:** General anesthesia, infection, nursing, respiratory tract, risk factor, surgery

## Abstract

**Objectives:**

We aimed to explore the incidence rate of respiratory tract infection (RTI) in patients after surgery under general anesthesia and to analyze its risk factors and nursing countermeasures.

**Methods:**

The clinical data of 136 patients treated from June 2020 to June 2022 were retrospectively analyzed. The incidence rate of RTI within 3 d after surgery was recorded. They were divided into an infection group and a non-infection group. The distribution of pathogenic bacteria was observed, and their clinical data.

**Results:**

RTI occurred in 16 of 136 cases (11.76%) after surgery under general anesthesia. Eighteen strains of pathogenic bacteria were cultured from these 16 cases, including Gram-negative bacteria in 13 cases (72.22%), Gram-positive bacteria in 4 cases (22.22%), and fungi in 1 case (5.56%). In the infection group, the proportions of patients with histories of smoking and lung surgery and orotracheal intubation were higher than those of the non-infection group, and the extubation time was later (P<0.05). History of smoking, orotracheal intubation, delayed extubation, and history of lung surgery were risk factors for RTI after surgery under general anesthesia (OR>1, P<0.05).

**Conclusion:**

History of smoking, orotracheal intubation, delayed extubation, and history of lung surgery may increase the risk of RTI after surgery under general anesthesia.

## Introduction

General anesthesia, as one of the commonly used anesthesia modes in clinical practice, can temporarily restrain the central nervous system of patients through intramuscular injection, intravenous injection, and respiratory tract inhalation of anesthetic drugs. It can effectively relieve intraoperative pain, improve perioperative safety, and cause hypoalgesia, drowsiness, and reflex inhibition^1^. However, a strong inhibitory effect of anesthetic drugs on the central nervous system and tracheal intubation may damage the respiratory tract of patients, increasing the risk of postoperative respiratory tract infection (RTI)^2^.

RTI is a common complication after surgery under general anesthesia, greatly influencing the patients' physical and mental health and prognosis. In recent years, the incidence rate of nosocomial RTI has been rising due to the gradual increase in the types, frequency, and dosage of antibacterial drugs,and the number of drug-resistant strains has also increased^3^,^4^. In addition, patients with surgical trauma are in a stress state and have weak defense ability, so they are prone to RTI in hospitals where a variety of pathogenic bacteria accumulate^5^. RTI in patients after surgery under general anesthesia may cause pulmonary inflammation or pulmonary heart disease with cardiac insufficiency, weaken human immune function, and affect the rehabilitation process^6^. Thus, it is of significance to explore the risk factors for RTI after surgery under general anesthesia and to take corresponding intervention measures for ameliorating the prognosis of patients. In this study, therefore, we explored the incidence rate of RTI in patients after surgery under general anesthesia and analyzed its risk factors and nursing countermeasures, aiming to provide references for RTI prevention and treatment.

## Materials and Methods

### General data

The clinical data of 136 patients undergoing surgery under general anesthesia in our hospital from June 2020 to June 2022 were retrospectively analyzed. There were 72 males and 64 females aged 48-72 years, with a mean of (59.37±6.25) years, and the body mass index (BMI) was 19-30 kg/m2, with a mean of (25.63±2.14) kg/m^2^.

### Inclusion and exclusion criteria

The inclusion criteria were as follows: (1) Patients undergoing surgery under general anesthesia, (2) those with normal coagulation function, (3) those with stable vital signs, and (4) those with complete clinical data.

The exclusion criteria were as follows: (1) Patients complicated with immune system dysfunction, (2) those with pre-existing RTI before surgery, (3) those complicated with severe malnutrition, (4) those complicated with multiple organ dysfunction such as liver or kidney dysfunction, (5) those complicated with malignancies or other severe diseases, or (6) those who had poor compliance with this study due to preoperative cognitive impairment or mental disorders.

### Assessment of RTI

The incidence of RTI was assessed within 3 days after surgery under general anesthesia in accordance with the diagnostic criteria for RTI7: with clinical symptoms and signs such as nasal congestion, cough, sneezing, runny nose and sore throat, dry and moist rales found by lung auscultation, and no abnormalities or increased lung markings in chest X-ray film.

### Detection of pathogenic bacteria

Respiratory tract secretion samples were harvested using an anti-pollution brush. Specifically, the brush protected by a double cannula and added polyethylene glycol at the distal opening was slowly inserted into the lower respiratory tract, extended to collect the respiratory tract secretion, withdrawn and placed in the collection bottle to obtain the sample stock solution. Later, the samples were cultured on a plate and counted.

### Collection of clinical data

At admission, the clinical data of patients were collected, including gender, BMI, age, history of smoking [yes or no, smoking index (daily cigarette consumption × years of smoking) >200 was considered a history of smoking], history of drinking (yes or no, mean daily alcohol consumption >40 g in men and >20 g in women for >1 month, or monthly drinking frequency ≥1 time for >6 months was considered a history of drinking), intubation route (nasal cavity or oral cavity), intubation mode (direct or blind), intraoperative blood transfusion (yes or no), anesthesia time, extubation time, history of lung surgery (yes or no), preoperative use of antibacterial drugs or hormones (yes or no), diabetes mellitus (yes or no, based on the diagnostic criteria for diabetes mellitus^8^), and hypertension (yes or no, based on the diagnostic criteria for hypertension^9^).

### Statistical analysis

SPSS23.0 software (IBM Inc., USA) was used for statistical analysis. The measurement data were described by (±s) and analyzed by the t-test. The count data were described by [n (%)] and analyzed by the χ2 test. The risk factors for RTI after surgery under general anesthesia were explored by logistic regression analysis. P<0.05 was considered statistically significant.

## Results

### Incidence rate of RTI

RTI occurred in 16 out of 136 cases (11.76%) after surgery under general anesthesia.

### Distribution of pathogenic bacteria in patients with RTI

A total of 18 strains of pathogenic bacteria were cultured from these 16 cases, including Gram-negative bacteria in 13 cases (72.22%), Gram-positive bacteria in 4 cases (22.22%), and fungi in 1 case (5.56%) (Table 1).

**Table 1:** Distribution of pathogenic bacteria in patients with RTI

Pathogenic bacteria		Strain (n)	Percentage (%)
Gram- negative bacteria	*Staphylococcus aureus*	2	11.11
	*Enterococcus*	1	5.56
	*Streptococcus*	1	5.56
Gram-positive bacteria	*Pseudomonas aeruginosa*	6	33.33
	*Klebsiella pneumoniae*	4	22.22
	*Acinetobacter baumannii*	3	16.67
Fungi	*Candida albicans*	1	5.56

### Clinical data

In the infection group, the proportions of patients with histories of smoking and lung surgery and receiving orotracheal intubation were higher than those in the non-infection group, and the extubation time was later than that in the non-infection group (P<0.05). There were no significant differences in gender, BMI, age, history of drinking, intubation mode, anesthesia time, preoperative use of antibiotics or hormones, diabetes mellitus, or hypertension between the two groups (P>0.05) (Table 2).

**Table 2:** Clinical data

Indicator		Infection group (n=16)	Non-infection group (n=120)	Statistical value	P
Gender [n (%)]	Male (n=72)	9 (56.25)	63 (52.50)	χ^2^ =0.0	0.77
	Female (n=64)	7 (43.75)	57 (47.50)	80	8
BMI (±*s*, kg/m^2^)		25.76±2.1	25.61±2.0	*t*=0.26	0.78
		9	8	9	8
Age (±*s*, year)		60.14±6.4	59.27±6.1	*t*=0.52	0.59
		3	8	7	9
History of smoking [n (%)]	Yes (n=37)	10 (62.50)	27 (22.50)	χ^2^ =9.4	0.00
	No (n=99)	6 (37.50)	93 (77.50)	75	2
History of drinking [n (%)]	Yes (n=30)	5 (31.25)	25 (20.83)	χ^2^ =0.3	0.53
	No (n=106)	11 (68.75)	95 (79.17)	88	3
Intubation route [n (%)]	Nasal cavity (n=102)	8 (50.00)	94 (78.33)	χ^2^ =4.6	0.03
	Oral cavity (n=34)	8 (50.00)	26 (21.67)	28	2
Intubation mode [n (%)]	Direct	7 (43.75)	68 (56.67)	χ^2^ =0.9	0.32
	Blind	9 (56.25)	52 (43.33)	52	9
Intraoperative blood transfusion [n (%)]	Yes	2 (12.5)	16 (13.33)	χ^2^ =0.0	0.76
	No	14 (87.50)	104 (86.67)	90	4
Anesthesia time (±*s*, h)		2.46±0.72	2.28±0.68	*t*=0.98	0.32
				8	5
Extubation time (±*s*, h)		3.12±0.67	2.07±0.53	*t*=7.20	<0.0
				7	01
History of lung surgery [n (%)]	Yes (n=11)	4 (25.00)	7 (5.83)	χ^2^ =4.6	0.03
	No (n=125)	12 (75.00)	113 (94.17)	36	1
Preoperative use of antibiotics or hormones [n (%)]	Yes (n=44)	6 (37.50)	38 (31.67)	χ^2^ =0.2	0.63
	No (n=92)	10 (62.50)	82 (68.33)	20	9
Diabetes mellitus [n (%)]	Yes (n=26)	3 (18.75)	23 (19.17)	χ^2^ =0.0	0.76
	No (n=110)	13 (81.25)	97 (80.83)	89	5
Hypertension [n (%)]	Yes (n=41)	5 (31.25)	36 (30.00)	χ^2^ =0.0	0.85
	No (n=95)	11 (68.75)	84 (70.00)	35	1

### Logistic regression analysis results of risk factors for RTI after surgery under general anesthesia

**Figure 1:**
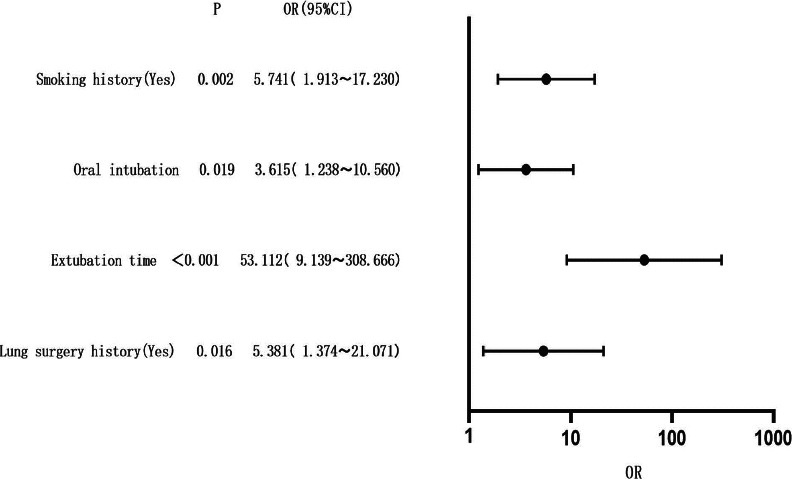
A forest plot of factor characteristics based on multivariate logistic regression analysis

Logistic regression analysis was conducted, with the incidence rate of RTI after surgery under general anesthesia as the dependent variable (1=yes, 0=no), and the indicators with significant differences (history of smoking, intubation route, extubation time, and history of lung surgery) as the independent variables. The assignment of independent variables is listed in Table 3. The results showed that history of smoking, orotracheal intubation, delayed extubation, and history of lung surgery were the risk factors for RTI after surgery under general anesthesia (odds ratio>1, P<0.05) (Table 4 and Figure 1).

**Table 3:** Assignment of independent variables

Independent variable	Description	Value assignment
History of smoking	Categorical variable	1= “Yes”, 0= “No”
Intubation route	Categorical variable	1= “Oral”, 0= “Nasal”
Extubation time	Continuous variable	-
History of lung surgery	Categorical variable	1= “Yes”, 0= “No”

**Table 4:** Logistic regression analysis results of risk factors for RTI after surgery under general anesthesia

Indicator	*β*	Standard error	*Wald* χ^2^	P	Odds ratio	95% confidence interval
History of smoking	1.7	0.561	9.71	0.00	5.741	1.913-17.230
	48		2	2		
Intubation route	1.2	0.547	5.52	0.01	3.615	1.238-10.560
	85		2	9		
Extubation time	3.9	0.898	19.5	<0.0	53.112	9.139-308.666
	72		73	01		
History of lung	1.6	0.696	5.83	0.01	5.381	1.374-21.071
surgery	83		9	6		

## Discussion

In this study, RTI occurred in 16 out of 136 cases (11.76%) after surgery under general anesthesia, suggesting a high incidence rate of RTI. Patients with RTI usually suffer from cough, fever, expectoration, suffocation, and even shock symptoms in severe cases, harming physical rehabilitation^10^. Therefore, it is crucial to prevent RTI following surgery under general anesthesia.

Additionally, we herein found that history of smoking, orotracheal intubation, delayed extubation, and history of lung surgery were the risk factors for RTI after surgery under general anesthesia. First, tobacco contains a large number of harmful substances such as tar, nicotine, carbon monoxide, and alkane. These harmful substances bind the tracheal, bronchial, and alveolar surface, and generate chemical and physical stimulation to long-term smokers, thus damaging the respiratory tract and immune defense functions^11^. Long-term smokers have significantly more pathogenic bacteria colonizing the respiratory tract than those in normal people^12^,^13^. Surgery under general anesthesia further destroys the defense function, making these patients more susceptible to RTI^14^. Second, a large number of pathogenic bacteria are present in the oral cavity^15^. However, patients undergoing surgery under general anesthesia are in poor physical conditions, so the bacteria easily enter the respiratory tract by orotracheal intubation to cause infection^16^. At the same time, tracheal intubation mechanically stimulates the oral and pharyngeal mucosae of patients and causes local mucosal injury, thus attenuating the respiratory barrier function and affecting its ability to filter pathogenic bacteria. As a result, a large number of respiratory tract secretions accumulate, and pathogenic bacteria directly enter the respiratory tract, increasing the infection risk^17^. In addition, gastric contents undergo reflux into the lower respiratory tract due to relaxation of the cardiac sphincter under anesthesia, raising the incidence rate of infection^18^. Endotracheal intubation can ensure an unobstructed airway during anesthesia, but the intubation tube is a foreign body, so long-term retention stimulates the surrounding tissues and induces inflammation^19^.

Third, a later extubation after surgery under general anesthesia leads to more severe damage to the respiratory tract mucosa of patients, then weakening the defense function and raising the risk of gastrointestinal reflux, so aspiration easily occurs and the incidence rate of RTI increases^20^,^21^. Fourthly, the lung function of patients with a history of lung surgery is damaged, causing repeated cough, dyspnea, and shortness of breath. As a consequence, the ciliary function of the respiratory tract is suppressed, and its ability to expectorate and defend against pathogenic microorganisms is weakened, making the respiratory tract vulnerable to pathogenic invasion and inducing RTI finally^22^,^23^.

The following intervention measures targeting the above risk factors were recommended. Patients with a history of smoking should actively quit smoking before surgery, improve lung function, and enhance the defense ability through moderate respiratory training and aerobic exercise to prevent postoperative RTI. Nasal intubation is recommended to minimize the irritation to oral and pharyngeal mucosae and to maintain the respiratory barrier function. Before intubation, strict oral cleaning and disinfection are required for patients receiving orotracheal intubation, thereby killing oral pathogenic microorganisms to prevent possible infection during tracheal intubation^24^. In addition, the patients should be strictly deprived of food and water before surgery to avoid the reflux of gastric contents during surgery. Early extubation should be carried out to reduce the risk of RTI. Moreover, ward disinfection, and oropharyngeal cleaning and nursing should be strictly performed for patients with a history of lung surgery, thus avoiding cross-infection. Meanwhile, sputum suction should be performed immediately once rales are heard.

In this study, Gram-negative bacteria were dominant in the pathogenic bacteria inducing RTI after surgery under general anesthesia, accounting for 72.22% of the total. Therefore, empirical anti-infective treatment should be conducted to inhibit disease progression in the first place once the symptoms and signs of RTI are found, and pathogen testing and drug sensitivity test should be timely carried out, based on which the therapeutic regimen can be adjusted to improve the rehabilitation of patients^25^. Moreover, elderly patients are physically vulnerable and may be complicated with various underlying diseases, so age is a risk factor for RTI after surgery under general anesthesia^26^,^27^. In this study, however, the patients enrolled in both groups were all old, showing no significant differences.

## Conclusion

History of smoking, orotracheal intubation, delayed extubation, and history of lung surgery may increase the risk of RTI after surgery under general anesthesia. In clinical practice, it is necessary to control the above risk factor and to provide targeted nursing countermeasures for reducing the incidence rate of RTI.
